# Acute Traumatic Tear of the Gluteus Medius and Gluteus Minimus in a Marathon Runner

**DOI:** 10.31486/toj.18.0090

**Published:** 2019

**Authors:** Brian Godshaw, Michael Wong, Connor Ojard, Gerard Williams, Misty Suri, Deryk Jones

**Affiliations:** ^1^ Ochsner Sports Medicine Institute, Ochsner Clinic Foundation, New Orleans, LA; ^2^ The University of Queensland Faculty of Medicine, Ochsner Clinical School, New Orleans, LA

**Keywords:** *Arthroscopy*, *buttocks*, *endoscopy*, *gluteus medius*, *gluteus minimus*, *sprains and strains*

## Abstract

**Background:** Tears of the gluteus medius and gluteus minimus are common causes of chronic lateral hip pain in the middle-aged population. These tears are postulated to occur after chronic degeneration of the muscle-tendon unit. The majority of these patients have a long history of peritrochanteric pain. Acute traumatic tear of the gluteus medius and gluteus minimus in otherwise asymptomatic patients is rare but can occur.

**Case Report:** We report the case of a 78-year-old male marathon runner with acute traumatic tear of the gluteus medius and gluteus minimus. After conservative management (physical therapy, a nonsteroidal antiinflammatory drug for pain, and cortico-steroid and local anesthetic injection) failed, the patient underwent operative repair. The surgery was successful, and the patient returned to his preinjury lifestyle 6 months postoperatively with no limitations.

**Conclusion:** In most cases, chronic injuries are far more common than acute tears. Because of the nonspecific and slowly progressive symptoms, patients are often misdiagnosed with radiculopathy, osteoarthritis, or trochanteric bursitis. Patients typically present to the clinic with an insidious onset of dull pain over the lateral hip. This pain is often worse when lying on the affected side. Certain gluteal-focused movements, such as climbing stairs, may exacerbate the pain. To our knowledge, our report is only the third case of acute traumatic tear of the gluteus medius and gluteus minimus reported in the literature.

## INTRODUCTION

Tears of the gluteus medius and gluteus minimus are common causes of chronic peritrochanteric pain, with gluteus medius tears affecting up to 25% of late-middle-aged women and 10% of middle-aged men.^1^ Because of their vague and often indolent symptoms, tears are often misdiagnosed and may be found incidentally during fracture fixation or hip arthroplasty.^2^ While the etiology remains unclear, tears of the gluteus medius and gluteus minimus are generally believed to be attributable to a degenerative process of the trochanteric enthesis, preceded by chronic lateral hip pain.^2,3^ Acute traumatic tears in asymptomatic patients are exceedingly rare, with very few cases reported in the literature.^2,4^ We report a case of traumatic tear of the gluteus medius and gluteus minimus and review relevant literature.

## CASE REPORT

A 78-year-old previously healthy male marathon runner presented with right lateral hip pain and groin pain radiating to the back. Two months prior, the patient was running with his dog when he sustained a forced external rotation injury to his right lower extremity. He felt a “pop,” followed by a sudden pulling pain in his groin. The initial pain resided, but his posterior hip remained sore, and he attributed it to a possible muscle strain. He had no history of injury or symptoms related to the hip or groin.

**Figure 1.**
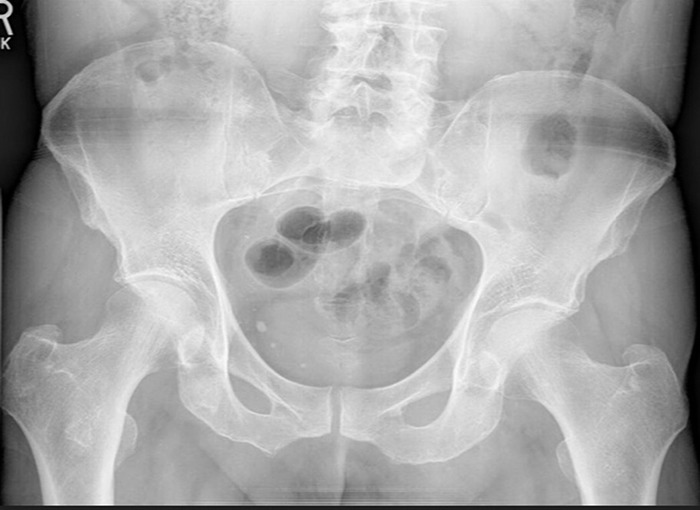
**Preoperative anteroposterior radiograph shows mild spurring of bilateral acetabulae with mild joint space narrowing.**

On physical examination, the patient had a nonantalgic gait. Lower extremities appeared normal with preserved range of motion. However, he reported pain with forced internal rotation and circumduction, as well as tenderness around the psoas tendon. Neurovascular examination was within normal limits. X-ray of the hip showed mild joint space narrowing and osteopenia (Figure 1). Right psoas strain was diagnosed. Initial management consisted of physical therapy and a nonsteroidal antiinflammatory drug (NSAID) for pain relief.

**Figure 2.**
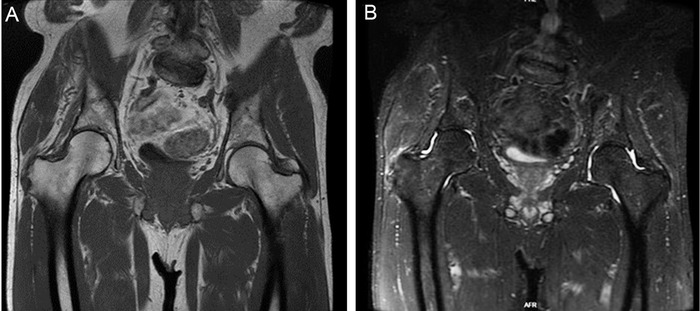
**T1 (A) and T2 (B) coronal magnetic resonance images show high-grade retracted tear of the right gluteus medius and gluteus minimus tendon.**

Ten months later, the patient returned to clinic with worsening of pain, especially in his lateral right hip. He stated that he completed physical therapy but had minimal relief. After therapy, he continued to run and completed a marathon. However, the pain increased to a debilitating point and forced him to quit running. On physical examination, he had preserved range of motion, with pain during hip abduction and tenderness over the right greater trochanter. Magnetic resonance imaging (MRI) of the right hip demonstrated a full-thickness tear of the right gluteus medius and gluteus minimus tendons at their greater trochanter insertion with 3.1 cm of retraction (Figure 2).

The patient was referred to pain management for a trial of injection containing 20 mg of triamcinolone and 1.5 mL of bupivacaine to the right hip adductor and right gluteus medius and gluteus minimus tendon sheaths under fluoroscopy. However, this treatment provided minimal relief. After the failure of conservative treatment modalities, the patient elected operative repair.

**Figure 3.**
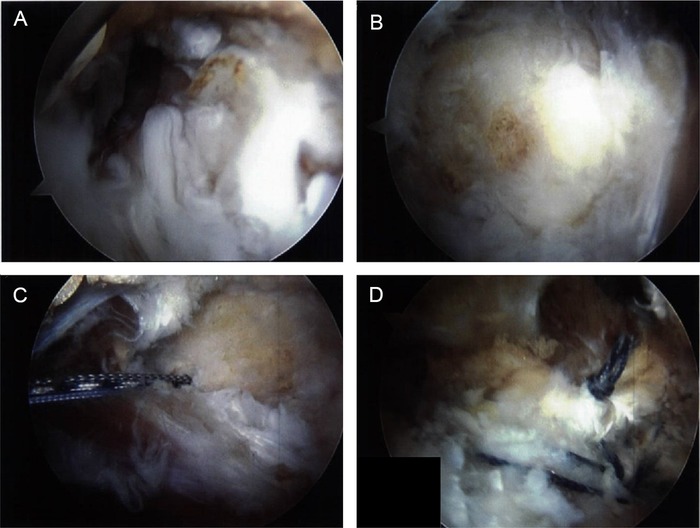
**Intraoperative arthroscopic images show (A) inflammation of the right trochanteric bursa, (B) vastus lateralis ridge after debridement, (C) triple-loaded anchor placed into the trochanteric area, and (D) final image of double row construct.**

**Figure 4.**
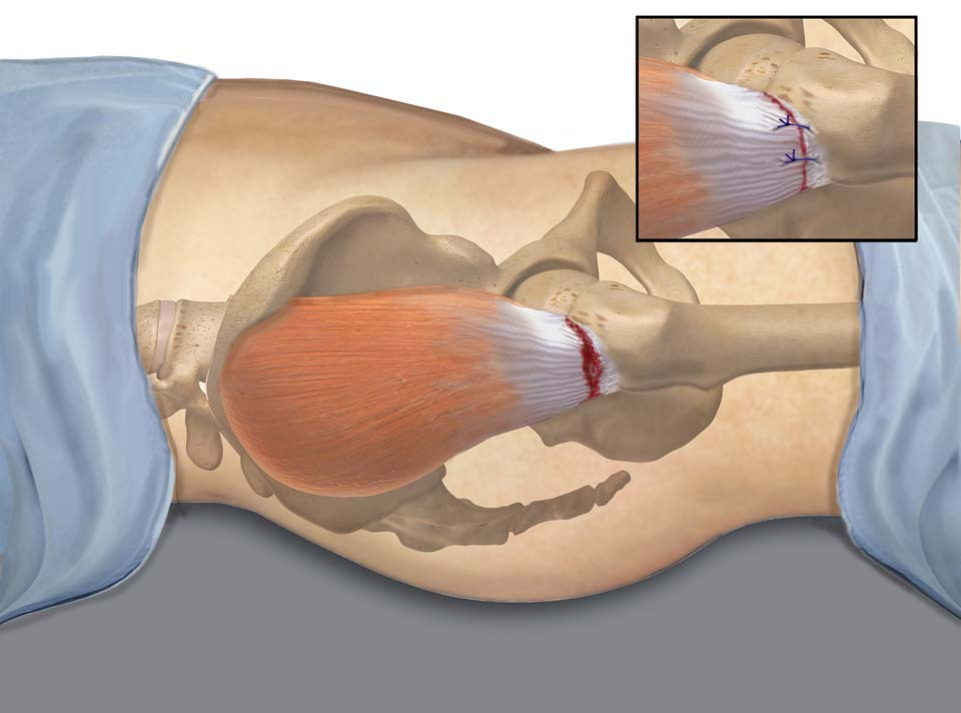
**Illustrations demonstrate tear and repair of the gluteus medius and gluteus minimus tendons.**

Arthroscopic technique was chosen. After the patient was appropriately positioned, proximal and distal anterior portals were established with fluoroscopic guidance. The trochanteric bursa was inflamed (Figure 3A) and was debrided (Figure 3B), exposing the gluteus medius and gluteus minimus tendon insertion points. Using a spinal needle and direct visualization with the arthroscope, a posterior viewing portal was established posterior to the trochanteric area, as well as an accessory portal over the vastus lateralis ridge. A 5.5 mm triple-loaded Healix Anchor (DePuy Synthes) was placed into the trochanteric area (Figure 3C). Sutures were passed using a Mitek ExpresSew (DePuy Synthes) and tied with a modified Roeder knot. The posterior limb of the sutures was further secured with a Healix Advance Knotless Anchor (DePuy Synthes) at the distal aspect of the vastus lateralis ridge. The process was repeated with the anterior limbs of the sutures, with a second Mitek Healix Advance Knotless Anchor in the posterior aspect of the vastus lateralis ridge (Figure 3D and Figure 4). A CLARIX regenerative matrix (Amniox Medical, Inc.) was placed over the repair site, and the repair was directly injected with CLARIX FLO (Amniox Medical, Inc.).

A Bledsoe brace was placed immediately. Weight bearing as tolerated was initiated immediately with the brace locked in extension for ambulation and unlocked from 0° to 90° at rest. Physical therapy was initiated 5 days postoperatively. Active abduction and strengthening were avoided for 6 weeks. At the patient's 6-week postoperative visit, he walked with no limp, the brace was discontinued, and hip strengthening was initiated. A more aggressive strengthening and home exercise program was initiated 3 months postoperatively. By the patient's 6-month postoperative visit, he was able to run 4 miles pain free. At 10.5 months postoperatively, the patient successfully completed a marathon with no pain or issues.

## DISCUSSION

The 3 gluteal muscles help form the muscle group known colloquially as the buttocks. From superficial to deep, these muscles are the gluteus maximus, gluteus medius, and gluteus minimus. The gluteus medius and gluteus minimus originate at the gluteal surface of the ilium and insert on the greater trochanter of the femur. Innervated by the superior gluteal nerve, the gluteus medius and gluteus minimus aid in hip abduction and internal rotation of the thigh and leg.

The incidence of gluteus medius and minimus tears peaks around the seventh decade of life, and tears are more common in women than in men.^3^ Tears of the gluteus medius and gluteus minimus manifest as chronic lateral hip pain, also known as greater trochanteric pain syndrome (GTPS). In a study of more than 3,000 middle-aged adults, Segal et al estimated the prevalence of unilateral GTPS to be 15% in women and 6.6% in men, with bilateral GTPS found in 8.5% of women and 1.9% of men.^5^ While GTPS encompasses any etiology that causes lateral hip pain, including unequal leg lengths or iliotibial band syndrome, gluteus medius and gluteus minimus pathologies are the most common cause.^6^ An MRI study published in 2001 revealed that 83% of patients with lateral hip pain had findings consistent with gluteus medius pathology.^7^

While the exact etiology of gluteus medius and gluteus minimus tears remains unknown, one proposed cause is chronic degeneration of the muscle-tendon unit.^8,9^ Over time, this degeneration can lead to tendinopathy and chronic lateral hip pain before ultimately tearing and retracting from the trochanteric attachment.

Acute traumatic tears are rare. A literature search yielded only two reported cases.^2,4^ In most cases, chronic injuries are far more common than acute tears. Because of the nonspecific and slowly progressive symptoms, patients are often misdiagnosed with radiculopathy, osteoarthritis, or trochanteric bursitis.^4,10-12^ In a French survey of 84 orthopedic surgeons, 45% were not aware that tears could occur, which may contribute to misdiagnosis.^13^

Patients typically present to the clinic with an insidious onset of dull pain over the lateral hip. This pain is often worse when lying on the affected side. Certain gluteal-focused movements, such as climbing stairs, may exacerbate the pain.^8^ On physical examination, patients often have reproducible tenderness on palpation of the greater trochanter.^14,15^ Weakness in abduction may manifest as Trendelenburg gait. Trendelenburg gait has a reported sensitivity of 72.7% and specificity of 76.9% in detecting gluteus medius pathology and is the most sensitive and specific physical test for gluteus medius pathology.^6,7^ Pain during resisted hip abduction has a similar sensitivity but a lower specificity (sensitivity of 72.7%, specificity of 46.2%). Another test is the single leg stance in which the patient stands on the affected leg for 30 seconds. Gluteus medius tendon pathology is indicated if the patient experiences trochanteric pain.^7^

Conventional radiography of the pelvis, using a standing anteroposterior view, should be the initial imaging study. The x-ray may rule out concurrent hip osteoarthritis, osteonecrosis, or femoral neck fracture. Radiographic signs of pathology of the gluteus medius and gluteus minimus include peritendinous edema, as well as contour irregularities >2 mm in the superolateral greater trochanter. In a study by Steinert et al, the sensitivity of radiographic changes for tendon pathology was 40% and the specificity was 94%.^16^ If x-rays are nondiagnostic and the patient continues to have refractory lateral hip pain, an MRI should be obtained. A complete tear could appear as a focal discontinuity of the tendon with retraction.^17^ Partial-thickness tears show a thickened tendon with increased signal intensity on T2-weighted images. Tendinosis appears as increased signal intensity on T2-weighted images with a normal-appearing tendon.^18^

Initial management is often nonoperative. Patients should refrain from strenuous activity, and NSAIDs may be prescribed for pain. Physical therapy directed to stretch and strengthen relevant muscle groups may reduce pain and improve mobility. Last, corticosteroid and local anesthetic injections to the trochanteric area may be used.^5,19^ Certain symptoms may predict the failure of nonoperative management. Chandrasekaran et al reported that loss of abduction and any deviation of gait pattern were predictors of failure of nonoperative management.^20^ Other factors such as lateral hip pain, duration of symptoms, and range of motion did not affect the likelihood that patients would require surgery. For patients who have refractory pain and/or impairment in performing the activities of daily living, operative management may be considered.^15^

Historically, tears of the gluteus medius and gluteus minimus were surgically repaired with an open transosseous approach or suture anchor repairs. Alternatively, with the growing popularity of minimally invasive surgery, an endoscopic approach may also be used to access the peri-trochanteric space. In the systematic review of repair options by Chandrasekaran et al, open and endoscopic gluteal repairs had similar patient-reported outcomes.^19^ Measurement of satisfaction, pain scores, and return of muscle strength were comparable in both approaches; however, open techniques had a higher complication rate. Specifically, re-tears were seen in 10 of 128 patients in the open repair group vs 0 of 40 patients in the endoscopic group. Other complications such as wound infections and hematoma were also significantly higher in patients who had open repairs.

Overall, the outcomes after operative repair are favorable regardless of surgical approach or technique. In a study by Walsh et al, only 5% of subjects had a normal walking ability before surgery, but 78% of their subjects recovered a normal gait by 6 months after surgery.^21^ Davies et al reported improvement in the mean visual pain scale in 90% of patients at 1-year follow-up, showing that the mean pain score dropped from 7 preoperatively to 2 postoperatively *(P<*0.05).^22^ Davies et al also reported that >84% of patients were satisfied with their results at 5-year follow-up, with 78% of subjects noting a subjective improvement in function.^23^

## CONCLUSION

Tears of the gluteus medius and minimus are common causes of lateral hip pain in middle-aged adults. These tears are believed to follow a gradual course, marked by chronic degeneration and subsequent hip pain, but acute tears are rare. Our patient, however, presented with an acute traumatic tear of the gluteus medius and gluteus minimus. He underwent operative repair and was able to return to his preinjury lifestyle 6 months postoperatively with no limitations. To our knowledge, our report is only the third case of acute traumatic tear of the gluteus medius and gluteus minimus reported in the literature.

## Notes

This article meets the Accreditation Council for Graduate Medical Education and the American Board of Medical Specialties Maintenance of Certification competencies for Patient Care and Medical Knowledge.

## References

[1] Domb BG, Gui C, Lodhia P How much arthritis is too much for hip arthroscopy: a systematic review. Arthroscopy. 2015 Mar;31(3):520-529. doi: 10.1016/j.arthro.2014.11.008. 25543247

[2] Stanton MC, Maloney MD, Dehaven KE, Giordano BD Acute traumatic tear of gluteus medius and minimus tendons in a patient without antecedant peritrochanteric hip pain. Geriatr Orthop Surg Rehabil. 2012 6;3(2):84-88. doi: 10.1177/2151458512441795. 23569702 PMC3598407

[3] Howell GE, Biggs RE, Bourne RB Prevalence of abductor mechanism tears of the hips in patients with osteoarthritis. J Arthroplasty. 2001 1;16(1):121-123. doi: 10.1054/arth.2001.19158. 11172282

[4] Bewyer D, Chen J Gluteus medius tendon rupture as a source for back, buttock and leg pain: case report. Iowa Orthop J. 2005;25:187-189. 16089095 PMC1888788

[5] Segal NA, Felson DT, Torner JC, et al; Multicenter Osteoarthritis Study Group. Greater trochanteric pain syndrome: epidemiology and associated factors. Arch Phys Med Rehabil. 2007 8;88(8):988-992. doi: 10.1016/j.apmr.2007.04.014. 17678660 PMC2907104

[6] Kagan A 2nd. Rotator cuff tears of the hip. Clin Orthop Relat Res. 1999 11;(368):135-140. 10613161

[7] Bird PA, Oakley SP, Shnier R, Kirkham BW Prospective evaluation of magnetic resonance imaging and physical examination findings in patients with greater trochanteric pain syndrome. Arthritis Rheum. 2001 9;44(9):2138-2145. 11592379 10.1002/1529-0131(200109)44:9<2138::AID-ART367>3.0.CO;2-M

[8] Tibor LM, Sekiya JK Differential diagnosis of pain around the hip joint. Arthroscopy. 2008 12;24(12):1407-1421. doi: 10.1016/j.arthro.2008.06.019. 19038713

[9] Robertson WJ, Gardner MJ, Barker JU, Boraiah S, Lorich DG, Kelly BT Anatomy and dimensions of the gluteus medius tendon insertion. Arthroscopy. 2008 2;24(2):130-136. doi: 10.1016/j.arthro.2007.11.015. 18237695

[10] Aepli-Schneider N, Treumann T, Müller U, Schmid L Degenerative rupture of the hip abductors. Missed diagnosis with therapy-resistant trochanteric pain of the hips and positive Trendelenburg sign in elderly patients [in German]. Z Rheumatol. 2012 1;71(1):68-74. doi: 10.1007/s00393-011-0919-y. 22286357

[11] LaBan MM, Weir SK, Taylor RS ‘Bald trochanter’ spontaneous rupture of the conjoined tendons of the gluteus medius and minimus presenting as a trochanteric bursitis. Am J Phys Med Rehabil. 2004 10;83(10):806-809. 15385792 10.1097/01.phm.0000140792.48248.49

[12] Lachiewicz PF Abductor tendon tears of the hip: evaluation and management. J Am Acad Orthop Surg. 2011 7;19(7):385-391. 21724917 10.5435/00124635-201107000-00001

[13] Cormier G, Berthelot JM, Maugars Y; SRO (Société de Rhumatologie de l'Ouest). Gluteus tendon rupture is underrecognized by French orthopedic surgeons: results of a mail survey. Joint Bone Spine. 2006 7;73(4):411-413. doi: 10.1016/j.jbspin.2006.01.021. 16762585

[14] Lustenberger DP, Ng VY, Best TM, Ellis TJ Efficacy of treatment of trochanteric bursitis: a systematic review. Clin J Sport Med. 2011 9;21(5):447-453. doi: 10.1097/JSM.0b013e318221299c. 21814140 PMC3689218

[15] Gordon EJ Trochanteric bursitis and tendinitis. Clin Orthop. 1961;20:193-202. 13707155

[16] Steinert L, Zanetti M, Hodler J, Pfirrmann CW, Dora C, Saupe N Are radiographic trochanteric surface irregularities associated with abductor tendon abnormalities*?* Radiology. 2010 12;257(3):754-763. doi: 10.1148/radiol.10092183. 20876391

[17] Cvitanic O, Henzie G, Skezas N, Lyons J, Minter J MRI diagnosis of tears of the hip abductor tendons (gluteus medius and gluteus minimus). AJR Am J Roentgenol. 2004 1;182(1):137-143. doi: 10.2214/ajr.182.1.1820137. 14684527

[18] Kingzett-Taylor A, Tirman PF, Feller J, Tendinosis and tears of gluteus medius and minimus muscles as a cause of hip pain: MR imaging findings. AJR Am J Roentgenol. 1999 10;173(4):1123-1126. doi: 10.2214/ajr.173.4.10511191. 10511191

[19] Chandrasekaran S, Lodhia P, Gui C, Vemula SP, Martin TJ, Domb BG Outcomes of open versus endoscopic repair of abductor muscle tears of the hip: a systematic review. Arthroscopy. 2015 10;31(10):2057-2067.e2. doi: 10.1016/j.arthro.2015.03.042. 26033462

[20] Chandrasekaran S, Vemula SP, Gui C, Suarez-Ahedo C, Lodhia P, Domb BG Clinical features that predict the need for operative intervention in gluteus medius tears. Orthop J Sports Med. 2015 Feb 20;3(2):2325967115571079. doi: 10.1177/2325967115571079. 26535383 PMC4555614

[21] Walsh MJ, Walton JR, Walsh NA Surgical repair of the gluteal tendons: a report of 72 cases. J Arthroplasty. 2011 12;26(8):1514-1519. doi: 10.1016/j.arth.2011.03.004. 21798694

[22] Davies H, Zhaeentan S, Tavakkolizadeh A, Janes G Surgical repair of chronic tears of the hip abductor mechanism. Hip Int. 2009 Oct-Dec;19(4):372-376. 20041385 10.1177/112070000901900412

[23] Davies JF, Stiehl JB, Davies JA, Geiger PB Surgical treatment of hip abductor tendon tears. J Bone Joint Surg Am. 2013 Aug 7;95(15):1420-1425. 23925748 10.2106/JBJS.L.00709

